# Outbreak of Marburg Virus Disease, Equatorial Guinea, 2023

**DOI:** 10.3201/eid3105.241749

**Published:** 2025-05

**Authors:** Stephanie Ngai, Egmond Samir Evers, Angela Katherine Lao Seoane, George Ameh, Julienne N. Anoko, Céline Barnadas, Mary J. Choi, Janet Diaz, Luca Fontana, Pierre Formenty, Ingrid Hammermeister Nezu, Frédérique Jacquerioz, John Klena, Henry Laurenson-Schafer, Olivier le Polain de Waroux, Anaïs Legand, Raquel Medialdea Carrera, Tatiana Metcalf, Joel Montgomery, Silvia Morreale, María E. Negrón, Justino Obama Nvé, Mitoha Ondo’o Ayekaba, Boris I. Pavlin, Trevor Shoemaker, Yaimara Torres Hernandez, Mabel Varona Venta, Emily Z. Gutierrez, Florentino Abaga Ondo Ndoho, on behalf of the Marburg Virus Disease Outbreak Response Working Group

**Affiliations:** World Health Organization, Geneva, Switzerland (S. Ngai, E.S. Evers, A.K. Lao Seoane, G. Ameh, J.N. Anoko, C. Barnadas, J. Diaz, L. Fontana, P. Formenty, I. Hammermeister Nezu, H. Laurenson-Schafer, O. le Polain de Waroux, A. Legand, R. Medialdea Carrera, T. Metcalf, S. Morreale, B.I. Pavlin, Y. Torres Hernandez); Centers for Disease Control and Prevention, Atlanta, Georgia, USA (M.J. Choi, J. Klena, J. Montgomery, M.E. Negrón, T. Shoemaker, E.Z. Gutierrez); Hôpitaux Universitaires de Genève, Geneva (F. Jacquerioz); Ministerio de Sanidad y Bienestar Social, Malabo, Equatorial Guinea (J.O. Nvé, M. Ondo’o Ayekaba, F. Abaga Ondo Ndoho); Brigada Médica Cubana, Malabo (M. Varona Venta).

**Keywords:** Marburg virus, viruses, zoonoses, Marburg virus disease, filoviruses, viral hemorrhagic fever, outbreak response, Equatorial Guinea

## Abstract

In February 2023, the government of Equatorial Guinea declared an outbreak of Marburg virus disease. We describe the response structure and epidemiologic characteristics, including case-patient demographics, clinical manifestations, risk factors, and the serial interval and timing of symptom onset, treatment seeking, and recovery or death. We identified 16 laboratory-confirmed and 23 probable cases of Marburg virus disease in 5 districts and noted several unlinked chains of transmission and a case-fatality ratio of 90% (35/39 cases). Transmission was concentrated in family clusters and healthcare settings. The median serial interval was 18.5 days; most transmission occurred during late-stage disease. Rapid isolation of symptomatic case-patients is critical in preventing transmission and improving patient outcomes; community engagement and surveillance strengthening should be prioritized in emerging outbreaks. Further analysis of this outbreak and a One Health surveillance approach can help prevent and prepare for future potential spillover events.

Marburg virus disease (MVD) is a severe infectious illness caused by 2 closely related viruses (Marburg virus [MARV] and Ravn virus, within the genus *Orthomarburgvirus*) of the family *Filoviridae* (*1*). Before 2023, at least 15 outbreaks of MVD had been identified; most involved sporadic or small numbers of cases (*2*). The 2 largest known outbreaks occurred during 1998–2000 in the Democratic Republic of the Congo (154 total cases) (*3*) and during 2004–2005 in northern Angola (252 confirmed and 374 total cases) (*4*,*5*).

MVD is characterized by the onset of nonspecific symptoms, typically including fever, headache, chills, fatigue, and myalgia, followed by a rapid progression to severe illness that may include nausea, vomiting, diarrhea, and hemorrhagic symptoms (*6*). Case-fatality rates (CFRs) range from 23% to 88%, and death often follows shock and multiorgan failure (*7*–*9*). Human-to-human transmission of MARV occurs through direct contact with blood or other bodily fluids of MVD patients, contaminated materials, and blood, fluids, or tissues from bodies of persons who have died from MVD (*9*).

Egyptian rousette fruit bats (*Rousettus aegyptiacus*) have been identified as a primary reservoir host for MARV (*10*–*12*). Outbreaks of MVD have been linked to exposure to mines or caves, where Egyptian rousettes typically roost (*13*), and have been found to be infected with MARV (*3*,*8*,*11*,*12*,*14*–*16*). Although models have identified that Equatorial Guinea falls within the zoonotic niche of MVD (*17*) and the virus has been identified in bat populations in neighboring Gabon (*18*), no previous outbreaks of filovirus disease (Marburg or Ebola disease) have been identified in Equatorial Guinea, and MARV has not been identified in humans in neighboring countries (*19*).

On February 7, 2023, the Ministry of Health and Social Welfare of Equatorial Guinea (MINSABS) was notified about a cluster of deaths with suspected hemorrhagic fever in 2 villages in Nsok Nsomo District, Kié-Ntem Province, on the border with Cameroon and Gabon. MINSABS sent blood samples from 10 persons from the cluster and identified through active case finding to 2 World Health Organization (WHO) collaborating centers for viral hemorrhagic fevers, Centre Interdisciplinaire de Recherches Médicales de Franceville in Gabon (8 samples) and Institut Pasteur Dakar (IPD) in Senegal (5 repeat and 2 additional samples). One of the additional samples, from a hospitalized patient in Ebibeyín District who died on February 10 with an unclear epidemiologic link to the cluster of deaths, tested positive for MARV by real-time reverse transcription PCR at IPD on February 12. The government of Equatorial Guinea declared an outbreak of MVD the following day. We describe the epidemiologic characteristics of the 2023 MVD outbreak in Equatorial Guinea.

## Methods

### Geographic Area of the Outbreak

We identified all cases within the mainland continental region of Equatorial Guinea, which is dominated by lush rainforests within the Congo Basin rainforest. The population is concentrated in urban areas, although the small geographic area lends itself to movement between districts. Economic windfall from oil production has funded substantial investment in the country’s infrastructure and road networks in recent years.

### Response Structure

The response to the outbreak in Equatorial Guinea was led by MINSABS, with support from national and international partners. A response structure was organized around key pillars, including coordination, surveillance and epidemiology, case management, laboratory, infection prevention and control (IPC), risk communication and community engagement, operational support and logistics, and finance and administration. Strategic decisions were made by the Political Committee for Health Emergencies, chaired by the Vice President of Equatorial Guinea.

The response structure was activated immediately after the outbreak declaration. The initial response coordination was based in Ebibeyín and later relocated to Bata, Equatorial Guinea’s largest city and economic hub, in mid-March, after the identification of a confirmed case in Bata with indication of earlier probable cases and secondary household transmission.

### Case Investigation and Contact Tracing

We used the WHO-recommended case definition (*20*) and later adapted it (Appendix), once all identified ongoing transmission was located in Bata, to emphasize human-to-human transmission over exposure to mines, caves, and wild animals and to reflect symptoms observed among confirmed MVD case-patients managed in the Marburg treatment center (MTC), including rash and back pain. An alert cell coordinated the management of alerts about possible suspected cases received through an established national hotline. Investigators used a standardized case investigation form, based on the WHO template (*21*), to collect information on patient demographics, clinical history and symptoms, exposure history, and patient movements during the potential infectious period. We conducted investigations prospectively for cases identified after the declaration of the outbreak and retrospectively for initial cases; those included interviews with families and community contacts of patients and health facility records, where available. Contacts of confirmed and probable cases (Appendix) were quarantined at home, and we followed them in person daily for 21 days after their last exposure. We managed contacts who had onset of symptoms during the follow-up period as suspected cases.

### Laboratory Testing

We collected whole blood for diagnostic testing from suspected case-patients and oral swab samples from deceased persons suspected of having MVD. After laboratory confirmation of the first confirmed case at IPD, we established a field laboratory in Ebibeyín within 1 week, with considerable support from partners, but were delayed in initiating laboratory testing until March 10. We consolidated the field laboratory with the Bome laboratory facility in Bata in mid-March, alongside the epicenter of the outbreak and the response coordination, to reduce laboratory testing turnaround time.

The laboratory in Ebibeyín used the BioFire FilmArray system (bioMérieux, https://www.biomerieux.com) using Warrior Panel test cartridges; the use of the cartridges is restricted to laboratories designated by the US Department of Defense. After the laboratory was relocated, the BioFire Global Fever Panel was used. All samples tested in Ebibeyín were retested in Bata by using the RealStar Filovirus Screen 1.0 RT-PCR kit (altona Diagnostics, https://altona-diagnostics.com) for result confirmation, according to manufacturer instructions.

### Patient Management

Suspected patients were hospitalized in designated temporary transit or treatment centers; confirmed patients were transferred and managed in a dedicated MTC, where they received supportive care (*22*,*23*). The initial isolation and treatment ward was established in Ebibeyín; after transmission was identified in Bata, an isolation and treatment ward was designated within Bata Regional Hospital. A dedicated MTC was opened in the Mondong INSESO Hospital in Bata on March 28 and had capacity to manage 16 patients. Subsequently, all confirmed MVD patients in the continental region and suspected MVD patients in Bata and those referred from other districts were managed at the Mondong MTC. Teams deployed from health facilities were trained to conduct safe and dignified burials for deceased patients.

### Data Management and Statistical Analysis

We entered case investigation forms into a standardized Excel database (Microsoft, https://www.microsoft.com). We conducted analyses by using R version 4.2.2 (The R Project for Statistical Computing, https://www.r-project.org) and produced maps by using ArcGIS (Esri, https://www.esri.com). We excluded from analyses 1 confirmed sample (collected in Kié-Ntem Province) that could not be linked to any patient data; we included all other confirmed and probable cases in analyses. We excluded missing or unknown information from calculations of proportions. We calculated the serial interval (the time between symptom onset dates of an infector–infectee pair) and the time from illness onset to hospitalization and death for case-patients with reliable information on exposure, illness onset, or both. Cases were categorized by number of days spent being symptomatic in the community before isolation, and we calculated the proportion of cases with possible or probable onward transmission.

MINSABS authorized the analysis and publication of these data. Analyses in this report were a retrospective review of data that were collected for surveillance and operational response purposes during the outbreak, outside of a research context; as such, no further ethical approval was required.

## Results

### Epidemiologic Description of the Outbreak

**Figure 1:**
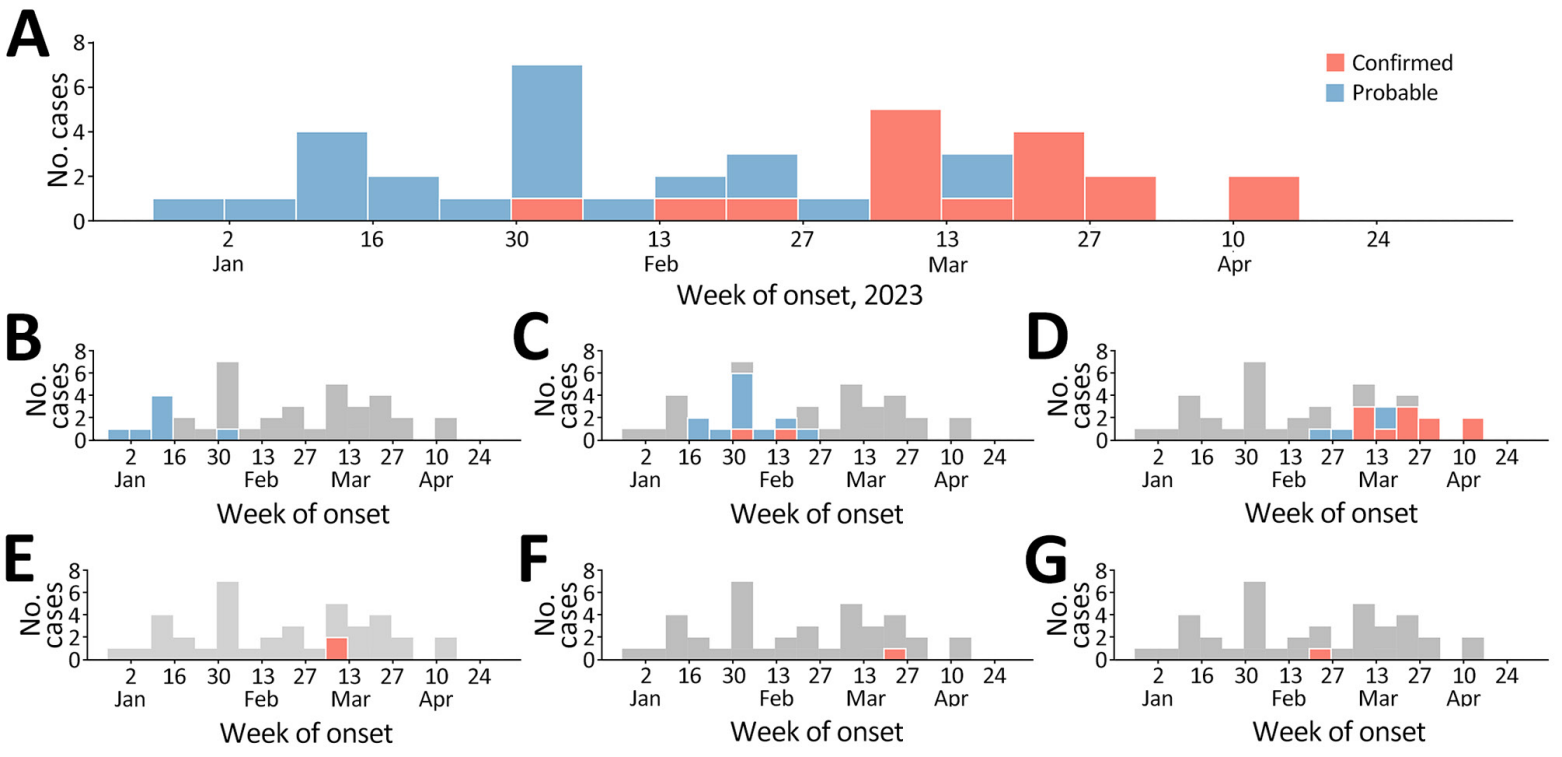
Confirmed and probable cases of Marburg virus disease, Equatorial Guinea, December 2022–April 2023. A) Confirmed and probable cases of Marburg virus disease, by week of illness onset and case classification. Where date of symptom onset was unavailable (1 case), estimated date of sample collection was used. B–G) Confirmed and probable cases of Marburg virus disease (as in shown in panel A), by district: B) Nsok Nsomo; C) Ebibeyín; D) Bata; E) Evinayong; F) Nsork; G) Unknown. Gray shading indicates total number of cases in the country.

**Figure 2:**
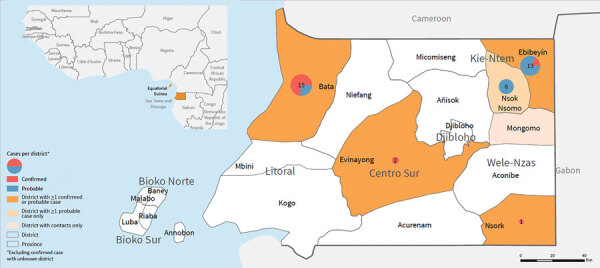
Confirmed and probable cases of Marburg virus disease, by district, Equatorial Guinea, January–April 2023.

We identified 16 laboratory-confirmed and 23 probable MVD cases during this outbreak. The onset date for symptoms of confirmed cases ranged from February 3 to April 19, 2023; the earliest identified probable case had an estimated onset of symptoms during the last week of December 2022 (Figure 1, panel A). We identified cases in 5 districts in 4 provinces: Bata (Litoral Province; 11 confirmed, 4 probable), Ebibeyín (Kié-Ntem Province; 2 confirmed, 11 probable), Nsok Nsomo (Kié-Ntem Province; 8 probable), Evinayong (Centro Sur Province; 2 confirmed), and Nsork (Wele-Nzas Province; 1 confirmed) (Figure 1, panel B–G; Figure 2). We recorded 166 alerts and listed 1,451 contacts.

**Figure 3:**
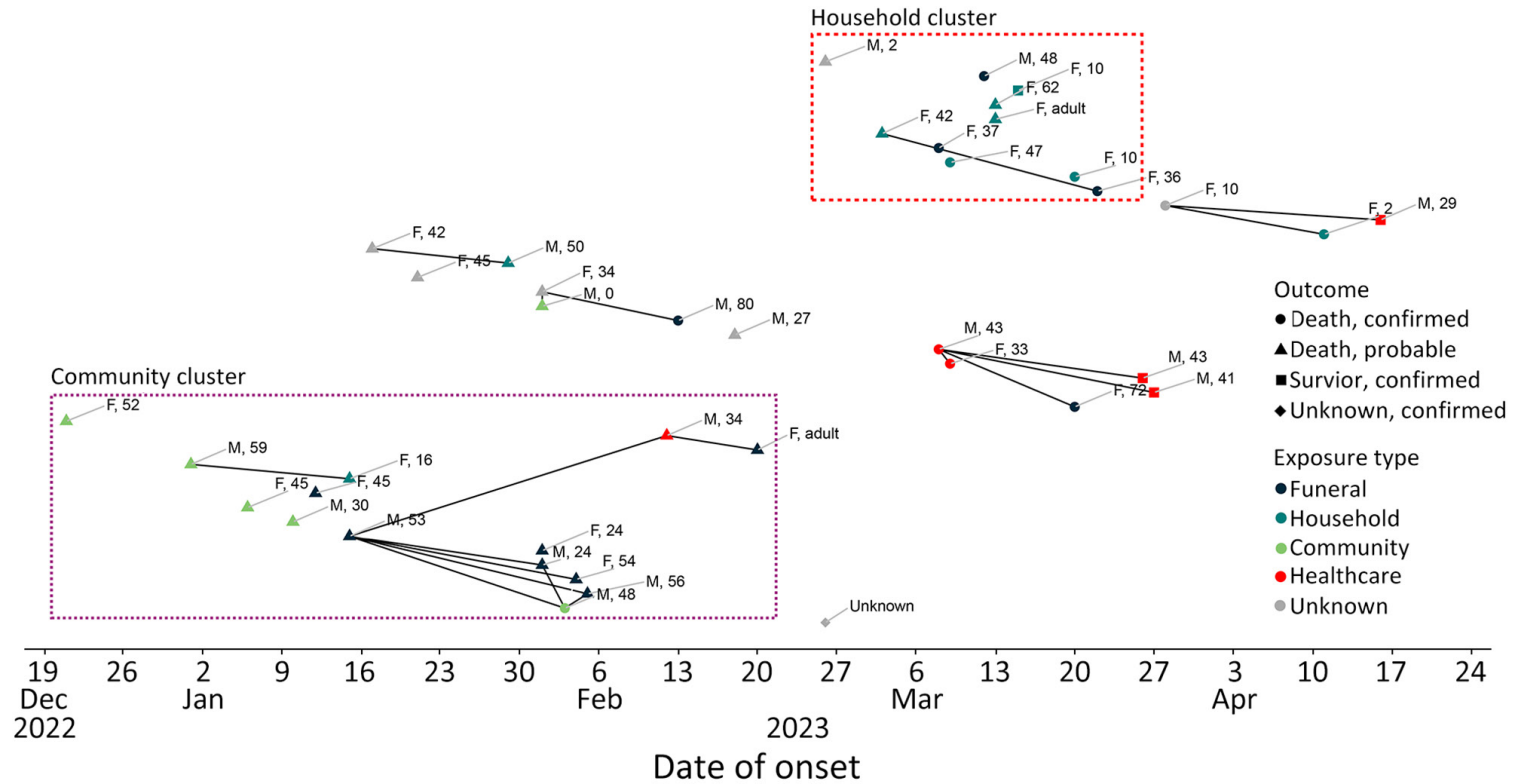
Chain of transmission of Marburg virus disease cases, by date of symptom onset and district, Equatorial Guinea, December 2022–April 2023. Where date of symptom onset was unavailable (1 case), estimated date of sample collection was used. Cases are labeled by sex and age in years. Solid black lines indicate known contact links suspected to be associated with transmission events. A degree of uncertainty is associated with some links shown. In the context of epidemiologically linked clusters with numerous contact links, infector–infectee pairs could not be determined in some cases. Boxes around cases indicate groups of cases with known epidemiologic links (i.e., clusters).

We identified 5 chains of transmission that could not be epidemiologically linked. Two clusters accounted for 60% of cases: the initial cluster of 14 cases in Nsok Nsomo and Ebibeyín Districts (35% of cases), linked to several funerals, and a household cluster of 10 cases in Bata District (25% of cases) (Figure 3). We identified smaller chains of transmission in the districts of Ebibeyín, Bata, and Evinayong (including 1 confirmed case in Nsork District and contacts in Nsork and Mongomo Districts). Initial genomic sequencing results from IPD suggest that all confirmed cases were linked to a single introduction of MARV into the population. Of the 16 confirmed case-patients, 9 (56%) were known contacts at the time of detection and 8 (50%) were identified during daily contact follow-up.

The median serial interval was 18.5 days (range 12–19 days) among 4 pairs of case-patients with known contact history. Among case-patients with reliable symptom onset date, the median time from symptom onset to case-patient isolation or burial was 8 days (n = 17; range 1–13 days); this period was shorter (4 days) among the subset of cases from Bata (n = 9; range 1–10 days). We observed onward transmission more frequently from case-patients who spent more time while symptomatic in the community: 58% (7/12) of case-patients who spent >5 days while symptomatic in the community had documented secondary transmission, compared with 20% (1/5) of case-patients who spent <5 days.

Participation in a funeral was a commonly reported risk factor for infection (56% [13/23]), as was contact with another case-patient in the same household (31% [12/39]). We identified 8 (21%) healthcare workers (HCWs): 5 confirmed and 3 probable case-patients, 1 of whom was a traditional healer. Five of the 8 HCWs died.

At least 3 confirmed case-patients sought care at private clinics after the onset of MVD. One of those clinics had poor IPC practices, and record keeping was minimal in a sample of clinics, complicating tracing of potential contacts. Although several probable case-patients visited traditional healers during their illness and we identified 1 probable MVD death in a traditional healer, we could not identify definitive transmission in these settings, which also had limited record keeping and are not included in routine health facility surveillance systems.

We suspect that all but 1 (who had other epidemiologic links) of the HCWs were infected through occupational exposure, although we only identified definitive exposure for 3 HCWs: infection followed invasive procedures (endotracheal intubation, IV insertion, and urinary catherization), with minimal or no personal protective equipment, performed on patients who were within hours of death and later confirmed to have MVD. An additional 2 HCWs with confirmed infection worked in the same service of a hospital and had illness onset within 1 day of one another, suggesting a common occupational exposure.

### Case Management

**Figure 4:**
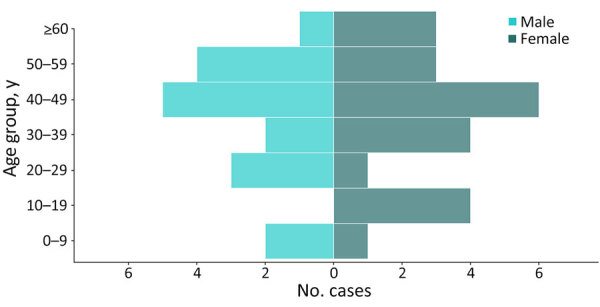
Age group and sex distribution of persons with confirmed or probable Marburg virus disease, Equatorial Guinea, January–April 2023.

Among case-patients with known outcomes, the CFR was 75% (12/16) among confirmed case-patients and 90% (35/39) among all case-patients. The median age of case-patients was 42 years (n = 37; range 7 months–80 years); 22 (56%) case-patients were female and 17 (44%) were male (Figure 4). We identified no pregnant women. The most frequently reported symptoms were fever (94% [32/34]), nausea or vomiting (79% [26/33]), and fatigue or general malaise (65% [22/34]). Diarrhea was reported in 13% (2/15) of confirmed and 83% (15/18) of probable case-patients, and hemorrhagic signs were reported in 57% (21/37) of all case-patients (Table).

**Table:** Reported symptoms among case-patients with Marburg virus disease, Equatorial Guinea, January–April 2023*

Symptom	Death, confirmed	Survivor, confirmed	Total, confirmed	Total, probable	Total, confirmed and probable
Fever	12/12 (100.0)	4/4 (100.0)	16/16 (100.0)	16/18 (88.9)	32/34 (94.1)
Abdominal pain	3/4 (75.0)	3/3 (100.0)	6/7 (85.7)	ND	6/7 (85.7)
Fatigue	8/12 (66.7)	4/4 (100.0)	12/16 (75.0)	10/18 (55.6)	22/34 (64.7)
Nausea/vomiting	8/11 (72.7)	2/4 (50.0)	10/15 (66.7)	16/18 (88.9)	26/33 (78.8)
Anorexia/loss of appetite	5/10 (50.0)	4/4 (100.0)	9/14 (64.3)	2/4 (50.0)	11/18 (61.1)
Convulsions	5/5 (100.0)	0/3 (0.0)	5/8 (62.5)	ND	5/8 (62.5)
Rash	0/2 (0.0)	3/3 (100.0)	3/5 (60.0)	ND	3/5 (60.0)
Any hemorrhagic sign	8/12 (66.7)	0/4 (0.0)	8/16 (50.0)	13/21 (61.9)	21/37 (56.8)
Joint or muscle pain	3/10 (30.0)	3/4 (75.0)	6/14 (42.9)	7/18 (38.9)	13/32 (40.6)
Headache	4/9 (44.4)	1/4 (25.0)	5/13 (38.5)	2/5 (40.0)	7/18 (38.9)
Conjunctivitis	2/3 (66.7)	0/3 (0.0)	2/6 (33.3)	ND	2/6 (33.3)
Hematemesis	4/11 (36.4)	0/4 (0.0)	4/15 (26.7)	13/18 (72.2)	17/33 (51.5)
Difficulty breathing	3/11 (27.3)	0/4 (0.0)	3/15 (20.0)	2/18 (11.1)	5/33 (15.2)
Difficulty swallowing	0/9 (0.0)	2/4 (50.0)	2/13 (15.4)	0/4 (0.0)	2/17 (11.8)
Bloody diarrhea	2/9 (22.2)	0/4 (0.0)	2/13 (15.4)	12/18 (66.7)	14/31 (45.2)
Diarrhea	2/11 (18.2)	0/4 (0.0)	2/15 (13.3)	15/18 (83.3)	17/33 (51.5)

*Values are no./no. (%). Denominators exclude case-patients where information on the symptom was unknown or not collected. ND, no data were available.

Among cases never managed in a designated MTC (confirmed case-patients with MVD diagnosed postmortem and probable case-patients), 77% (20/26) sought healthcare in a hospital setting before death. The median time from illness onset to initial hospitalization among hospitalized case-patients with reliable information on date of illness onset was 4 days (n = 15; range 1–9 days).

No confirmed case-patients were managed in the Ebibeyín treatment ward; 5 confirmed case-patients were admitted to the isolation unit in Bata Regional Hospital, of whom 1 survived. The Mondong MTC managed 5 confirmed case-patients, of whom 3 survived. The 4 surviving case-patients were admitted soon after illness onset (median 1 day; range 1–2 days), and the median time from onset to recovery was 14 days (range 10–15 days). Among deceased confirmed case-patients, the median time from illness onset to hospitalization was 6 days (n = 9; range 4–9 days); those case-patients admitted to a treatment center died shortly after admission (n = 7; median 2 days; range 1–4 days).

## Discussion

This outbreak of MVD in Equatorial Guinea had 39 identified confirmed and probable cases across 5 districts, plus 1 confirmed sample from a patient who was never identified. Transmission was concentrated in family clusters and often involved contact with deceased case-patients; more than half of all case-patients had a known household or funeral exposure. The mechanisms of community transmission events were not described for most cases, and the demographic distribution, with no clear overrepresentation of adult female case-patients, does not support caregiving as the principal risk factor (*4*,*5*). We never identified the initial case and exposure, but no case-patients reported exposures to bats or visits to mines (*3*).

Rapid isolation of symptomatic case-patients remains critical: those who survived sought care sooner at the MTC than did those who died. Three of the 4 survivors were HCWs and sought care immediately after symptom onset because of their familiarity with MVD. Case-patients who were isolated quickly after symptom onset also contributed less frequently to downstream transmission, and the relatively long serial interval among patients with reliable data suggests that most documented transmission occurred during late-stage disease. Those improved outcomes and decreased transmission risks underscore the importance of strong risk communication, community engagement, contact tracing programs, and early care in improving patient outcomes and preventing secondary transmission.

The number of unconnected chains of transmission raised concerns about undetected community circulation and highlighted the need for additional case-finding strategies. Many case-patients sought healthcare in hospital settings but did not have MVD diagnosed. Although most case-patients were managed in a hospital at some point during their illness, for most, no secondary nosocomial transmission was documented. However, limited IPC capacities combined with care-seeking behavior indicate a high potential risk for transmission. Although not observed during this outbreak, vertical transmission has been documented during other filovirus disease outbreaks (*24*), as has viral persistence in the placenta after recovery (*25*). Improved hospital record keeping would aid case finding and contact tracing, particularly for unconnected chains of transmission. The identified cases of nosocomial transmission occurred among HCWs who had inadequate protection and high-risk exposures to severely ill patients; better IPC standards, particularly in the midst of an outbreak, might have prevented these infections. Healthcare exposures were not limited to hospital settings; care seeking involved both traditional healers and small, low-cost private clinics. The role of small neighborhood clinics and traditional healers with inadequate IPC measures remains important in terms of limiting healthcare-related exposures and the potential as foci of infection for other patients in future outbreaks.

Fewer case-patients reported so-called wet symptoms than is typically expected during an outbreak of filovirus disease: only 13% of confirmed case-patients reported diarrhea, in contrast to 46% of a subset of confirmed case-patients from the 2004–2005 outbreak in Angola (*4*,*5*). Although information on nausea and vomiting was collected jointly, many confirmed case-patients reported nausea without vomiting. Wet symptoms were more frequently reported among probable cases, with 83% reporting diarrhea, although probable cases are representative of late-stage disease and this elevated prevalence might be partially attributable to recall bias during retrospective investigations. Among confirmed case-patients, convulsions and hemorrhagic signs were observed only in the hours preceding death. A case series further describes the clinical and laboratory progression of the 5 confirmed case-patients managed at the Mondong MTC (*23*). Case-patients often were positive for malaria at the time of MVD diagnosis; the high malaria prevalence and diversity of malaria species in Equatorial Guinea underscores the need to enhance malaria treatment during MVD outbreaks to avoid unnecessary confusion with MVD.

Although the origin of the outbreak was not identified, zoonotic spillover is the most likely route of infection, considering close proximity of the initially affected communities to wild bats and widespread consumption of wild animals, including bats (*26*). Genomic sequencing of the virus isolated from the first confirmed case-patient in this outbreak found that the isolate was most closely related to MARVs isolated from Egyptian rousettes in Sierra Leone (*27*), which in turn were similar to isolates collected from humans during the 2004–2005 outbreak in Angola (*28*). Detailed genomic sequencing results on samples from the Equatorial Guinea outbreak are not available; integration of additional sequencing results with epidemiologic data could further elucidate details on and connect chains of transmission.

We encountered several challenges during the response. Because there had not been a previous response to a filovirus disease outbreak in the country, the government of Equatorial Guinea requested support from some international partners, but simultaneous spread to multiple districts stretched resources thin from both the government and partners with presence in the country. After a delay between identifying and reporting initial cases, processes were established for regular information sharing. Because of limited existing field epidemiology and outbreak investigation capacity, along with community resistance, few alerts were triggered, documentation of alerts was incomplete, and identification of contacts was limited, particularly early in the response. Mortality surveillance, including systematic sampling of deaths, was not implemented. Weak existing disease surveillance and data management systems precluded further epidemiologic analyses to inform response activities. Inadequate risk perception was partly addressed through countrywide risk communication and community engagement efforts, although the lack of familiarity with filovirus diseases, severity of the cases, and tendency of the virus to infect entire families contributed to ongoing concerns among the population that MVD was related to witchcraft. We encountered challenges in implementing standardized contact definitions; during initial investigations, entire villages in which case-patients resided were listed as contacts. Poor communication and widespread enforcement of quarantine probably caused reluctance to engage with contact tracing and educational messaging. Standard IPC measures were limited; a national IPC program was established, although implementation was delayed at several facilities. Remdesivir was provided to 4 patients at the MTC on a compassionate use basis (*23*), but other potential treatments and candidate vaccines for MVD were not approved for use at the time of the outbreak.

Some data in this report, in particular those from initial investigations, are incomplete; we discarded values that were missing, unknown, or thought to be unreliable, resulting in small sample sizes. Information on clinical manifestations, particularly for probable case-patients, might not be reliable, because medical records were infrequently available and symptom information was ascertained during retrospective investigations. Information on risk factors was not complete for all case-patients. Retrospective identification of probable case-patients was biased toward identification of deaths; that bias might have inflated the CFR because nonfatal cases, particularly early in the outbreak, might have been missed. Individual-level contact tracing data were not entered into centralized databases and were not available for decision-making or analysis. We identified several unlinked chains of transmission, spanning multiple generations of transmission; we could not further analyze transmission dynamics. Laboratory data were not systematically linked to case investigation data and were not available for analysis.

The government declared the end of the MVD outbreak in Equatorial Guinea on June 8, 2023, nearly 4 months after its initial detection. The epidemiology of this outbreak adds to the limited knowledge about MVD, most of which is based on data from 2 large outbreaks with distinct epidemiologic characteristics (*3*–*5*), and reinforces the importance of early detection of cases to prevent transmission and improve outcomes for patients. The identification of this outbreak follows an increasing trend in the number of detected filovirus outbreaks (*29*–*31*). The unknown origin of the outbreak underscores the importance of incorporating a One Health approach to strengthening surveillance systems to understand the history of this outbreak and to prevent and prepare for future spillover events. Further investigation and seroprevalence studies should be considered to determine the number of unidentified cases and geographic areas to target for enhanced surveillance for MVD and other filovirus diseases. Overall limited International Health Regulations core capacities before the outbreak, which were assessed during the most recent WHO Joint External Evaluation and became apparent during the course of the outbreak, underscore the need for a WHO National Action Plan for Health Security for the country to develop its capacity to prevent, detect, and adequately respond to future health threats.

Appendix Additional information about outbreak of Marburg virus disease, Equatorial Guinea, 2023.
