# Supplementary material for: Outbreak of Marburg Virus Disease, Equatorial Guinea, 2023
Source: Emerg Infect Dis. 2025 May;31(5):887–95. doi: 10.3201/eid3105.241749 (PMC12044228; doi:10.3201/eid3105.241749)
Supplement: Appendix — Additional information about outbreak of Marburg virus disease, Equatorial Guinea, 2023. [file 24-1749-Techapp-s1.pdf]

Article DOI: https://doi.org/10.3201/eid3105.241749

EID cannot ensure accessibility for supplementary materials supplied by authors. Readers who have difficulty accessing supplementary content should contact the authors for assistance.

Outbreak of Marburg Virus Disease, Equatorial Guinea, 2023

Appendix

Case Definition

Marburg virus disease case definition, following modifications to the WHO recommended case definition for Ebola and Marburg virus diseases, implemented 17 April 2023.

Suspected case

- Any person with a sudden onset of ≥1 symptom indicative of Marburg virus disease (MVD) (fever, back pain, muscle pain, abdominal pain, loss of appetite, nausea or vomiting, fatigue, rash, difficulty swallowing or breathing, headache, diarrhea, hiccups) and a history of [one or more of the following]:
  - o Contact with a confirmed or probable case-patient of MVD
  - o Participation in a burial of an unexplained or sudden death
  - o Visiting >1 health care facility in the preceding 7 days, or
- Any person with sudden onset of fever and ≥3 symptoms indicative of MVD, or
- Any person with sudden onset of unexplained hemorrhage, convulsions, altered consciousness, or shock, or
- Anyone with unexplained and sudden death.

Probable case

- Any suspected case-patient evaluated by a clinician, or
- Any suspected case-patient that had died (and for which it was not possible to obtain a biologic sample) with an epidemiologic link to a confirmed case-patient.

Confirmed case

Any suspected or probable case-patient with a positive PCR or antibody laboratory result for Marburg virus.

Noncase

Any suspected or probable case-patient with a negative laboratory result for Marburg virus from a sample collected during the appropriate period while the case-patient was symptomatic. Case-patients who received a negative laboratory result from a sample collected within the first 72 hours following symptom onset were re-tested at least 72 hours following symptom onset.

Contact

Any person who, following the onset of a Marburg virus disease case-patient's symptoms:

- Slept in the same house as a case-patient, or
- Had direct physical contact with a case-patient or their bodily fluids during their illness or at their funeral, or
- Touched clothing or bed linens used by the case-patient, or
- Was breastfed by the case-patient, or
- Had contact with a case-patient or their samples in a health care or laboratory facility without appropriate personal protective equipment.
